# Quercetagetin Ameliorates Heat Stress-Induced Intestinal Damage via Oxidative Stress, Inflammation and Gut Microbiota in Mice

**DOI:** 10.3390/microorganisms14040896

**Published:** 2026-04-16

**Authors:** Xiuqiong Huang, Mingcan Wang, Zhixing Qing, Jianguo Zeng

**Affiliations:** 1 College of Food and Chemical Engineering, Shaoyang University, Shaoyang 422001, China; huangxqiong@163.com; 2 Yuelushan Laboratory, Changsha 410125, China; yichun202409@163.com; 3 Hunan Key Laboratory of Traditional Chinese Veterinary Medicine, Hunan Agricultural University, Changsha 410128, China; 4 College of Life Science and Environmental Resources, Yichun University, Yichun 336000, China

**Keywords:** oxidative stress, inflammation, gut microbiota, Nrf2 pathway, intestinal barrier, heat shock proteins, flavonol

## Abstract

Quercetagetin (QG), a principal flavonol from marigold (*Tagetes erecta* L.), is recognized for its potent antioxidant properties. However, its efficacy in mitigating intestinal injury under heat stress (HS) conditions remains unclear. We investigated the protective effects of QG using a mouse model of HS (41 °C, 70% humidity). Mice received oral QG (100 mg/kg/day) or saline for seven consecutive days before and during HS exposure. We assessed jejunal histopathology, oxidative stress markers, inflammatory cytokines, gene expression, and gut microbiota composition via 16S rRNA sequencing. QG supplementation significantly ameliorated HS-induced jejunal damage. It enhanced the activities of superoxide dismutase (SOD) and catalase (CAT) while reducing malondialdehyde (MDA) and pro-inflammatory cytokines (IL-1β, IL-6, TNF-α). QG downregulated the mRNA expression of heat shock proteins (Hsp70, Hsp90) and upregulated antioxidant-related genes (*SOD1*, *GPX4*, *CAT*, *NQO1*, *Nrf2*). Furthermore, QG preserved intestinal barrier integrity by upregulating tight junction proteins (*Occludin*, *Zo-1*, *Claudin*). 16S rRNA analysis revealed that QG significantly reshaped the gut microbiota, marked by an increased relative abundance of Lactobacillus and a decrease in potentially harmful taxa such as Allobaculum, Oscillibacter, and Colidextribacter. QG effectively alleviates HS-induced intestinal injury by enhancing antioxidant capacity, suppressing inflammation, and modulating the gut microbiota. These findings provide a scientific basis for the potential application of QG as a functional feed additive to improve animal health under heat stress conditions.

## 1. Introduction

Extreme high-temperature environments expose animals to heat stress (HS), triggering non-specific physiological responses that lead to oxidative stress, metabolic dysregulation, and intestinal dysfunction, ultimately compromising tissue integrity and productive performance [1,2]. The gastrointestinal tract is particularly vulnerable, as HS disrupts intestinal barrier function, promotes inflammatory signaling, and alters gut microbial homeostasis, thereby exacerbating systemic inflammation and metabolic disturbances [3,4]. Therefore, identifying effective and safe nutritional strategies to mitigate HS-induced intestinal damage is of paramount importance for sustaining animal health and agricultural productivity.

At the cellular level, HS activates the heat shock response (HSR), a highly conserved protective mechanism primarily governed by heat shock factor 1 (HSF1). HSF1 triggers the expression of molecular chaperones known as heat shock proteins (HSPs), such as HSP70 and HSP90, which assist in proper protein folding and prevent aggregation under stress [5,6]. Concurrently, HS elevates the production of reactive oxygen species (ROS), leading to oxidative damage [7]. The nuclear factor erythroid 2-related factor 2 (Nrf2) pathway is the master regulator of the cellular antioxidant defense. Upon activation, Nrf2 translocates to the nucleus and promotes the expression of antioxidant enzymes like NQO1, HO-1, SOD, and GPX [8,9,10]. Both the HSF1-HSP and Nrf2-ARE pathways are critical endogenous protective mechanisms that facilitate cellular adaptation and survival under HS [11]. The intricate crosstalk between these pathways is an area of active research [12,13].

Beyond cellular signaling, HS compromises the physical intestinal barrier by reducing blood flow, inducing oxidative stress, and downregulating tight junction proteins (*Occludin*, *Claudins*, *ZO-1*), leading to increased intestinal permeability [14,15,16]. This disruption is closely linked to alterations in the gut microbiota. A stable and balanced microbial community is essential for maintaining gut health [17,18]. HS can disrupt this equilibrium, often characterized by a reduction in beneficial bacteria such as Lactobacillus and an expansion of potentially pathogenic taxa [19,20,21,22]. This dysbiosis further aggravates barrier dysfunction and systemic inflammation.

Plant-derived extracts, valued for their bioactivity and low toxicity, offer a promising source of interventions against HS [23,24]. Quercetagetin (QG), a flavonol and a major bioactive component of marigold (*Tagetes erecta* L.), exhibits strong in vitro antioxidant activity [25,26]. Structurally similar to the well-studied quercetin, which possesses diverse pharmacological properties [27,28], QG may offer comparable or enhanced benefits. However, its potential to protect against HS-induced intestinal injury in vivo remains largely unexplored.

Consequently, this study was formulated to examine the protective effects of QG on HS-induced intestinal injury in a murine model. We postulated that supplementation with QG may alleviate HS-induced intestinal damage by augmenting antioxidant capacity, attenuating inflammation, and modulating gut microbiota, possibly through the regulation of the Nrf2 and HSF1 pathways. To evaluate this hypothesis, we assessed intestinal morphology, markers of oxidative stress, inflammatory cytokines, the expression of genes associated with antioxidant defense and barrier function, and the composition of gut microbiota using 16S rRNA sequencing.

QG effectively alleviates HS-induced intestinal injury by enhancing antioxidant capacity, suppressing inflammation, and modulating the gut microbiota. These findings provide a scientific basis for the potential application of QG as a functional feed additive to improve animal health under heat stress conditions.

## 2. Materials and Methods

### 2.1. Reagents and Chemicals

Quercetagetin (QG, purity > 89%, CAS: 90-18-6, batch No.: 3-0270-200717) was provided by Hebei Chenguang Biotechnology Co., Ltd. (Handan, China). Total protein concentration was determined using a BCA kit (Thermo Fisher Scientific Inc., Rockford, IL, USA). Biochemical and ELISA kits were purchased from the Nanjing Jiancheng Bioengineering Institute (Nanjing, China).

### 2.2. Animals and Experimental Design

Six-week-old specific-pathogen-free (SPF) male ICR mice (24 ± 2 g) were obtained from Changsha Tianqin Biotechnology Co., Ltd. (Changsha, China; license No. SCXK(Xiang)2019-0014). All experimental procedures were approved by the Ethics Committee of Hunan Agricultural University, China (No. 43321543). Mice were housed (6 per cage) under controlled conditions (24 ± 2 °C, 50 ± 10% humidity, 12 h light/dark cycle) with ad libitum access to standard chow and water. After one week of acclimatization, 18 mice were randomly divided into three groups (*n* = 6/group):

Normal Control (NC): Received oral saline and was kept under thermoneutral conditions.

Heat Stress (HS): Received oral saline and was subjected to heat stress.

Quercetagetin (QG): Received oral QG (100 mg/kg body weight/day) and was subjected to heat stress.

**Figure 1:**
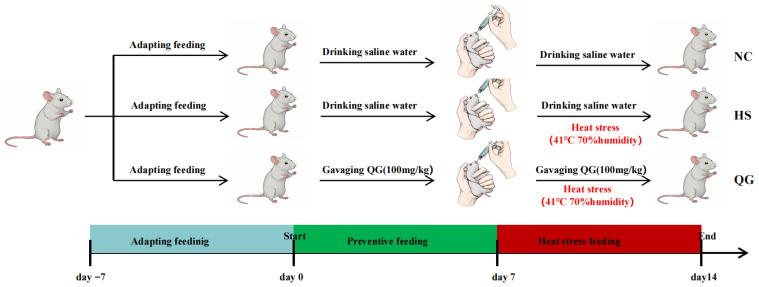
Schematic representation of the experimental design.

Mice in the QG and HS groups were gavaged daily with QG or an equal volume of saline, respectively, for 7 consecutive days. From day 1 to 7, mice in the HS and QG groups were exposed to heat stress (41 °C, 70% humidity) for 2 h daily (11:00 a.m. to 1:00 p.m.) [29]. The NC group was maintained under thermoneutral conditions. The experimental timeline is illustrated in Figure 1. Two hours after the final HS exposure on day 7, all mice were euthanized by cervical dislocation. Jejunum tissues were collected, snap-frozen in liquid nitrogen, and stored at −80 °C for subsequent analyses. Cecal contents were also collected for microbiota analysis.

### 2.3. Histopathological Examination of the Jejunum

Segments of the jejunum were preserved in 4% paraformaldehyde, subsequently embedded in paraffin, sectioned at a thickness of 5 µm, and stained with hematoxylin and eosin (H&E) as described in reference [30]. Pathological alterations were examined using a light microscope. Measurements of villus height and crypt depth were conducted on a minimum of 10 well-oriented villi per sample, utilizing ImageJ software (version 1.51) [31].

### 2.4. Measurement of Oxidative Stress and Inflammatory Markers in Jejunal Tissue

Jejunal tissue samples were homogenized in ice-cold saline to prepare a 10% (*w*/*v*) homogenate. After centrifugation (4 °C), the supernatant was collected. Commercial kits were used to measure the levels of malondialdehyde (MDA, Catalog No. A003-1-2), and the activities of superoxide dismutase (SOD, Catalog No. A001-3-2), glutathione peroxidase (GSH-Px, Catalog No. A005-1-2), and catalase (CAT, Catalog No. A007-1-1), following the manufacturer’s protocols (Nanjing Jiancheng Bioengineering Institute, Nanjing, China) [32]. The concentrations of heat shock protein 70 (HSP70, Catalog No. H264-2-2), cortisol (CORT, Catalog No. H205-1-2), tumor necrosis factor-alpha (TNF-α, Catalog No. H052-1-2), interleukin-1 beta (IL-1β, Catalog No. H002-1-2), and interleukin-6 (IL-6, Catalog No. H007-1-2) were quantified using ELISA kits (Nanjing Jiancheng Bioengineering Institute, Nanjing, China). All results were normalized to the total protein concentration, determined by a BCA assay (Catalog No. A045-3-2) [33].

### 2.5. RNA Extraction and Quantitative Real-Time PCR (qRT-PCR)

Total RNA was isolated from jejunum tissue using TRIzol reagent (Thermo Fisher Scientific, Catalog No. 15596026, Waltham, MA, USA). RNA (1 µg) was reverse-transcribed into cDNA using the HiScript 1st Strand cDNA Synthesis Kit (Accurate Biology, Changsha, China; Catalog No. R111-01). qPCR was performed on a qTOWER3 G Real-Time PCR System (Analytik, Jena, Germany) using SYBR Green Master Mix [34]. The thermal cycling conditions were: 95 °C for 30 s, followed by 40 cycles of 95 °C for 10 s and 60 °C for 30 s. Relative gene expression was calculated using the 2^−ΔΔCt^ method [35], with Gapdh as the internal control. Primer sequences are listed in Table 1 [36].

### 2.6. 16S rRNA Gene Sequencing and Bioinformatics Analysis of Gut Microbiota

Total genomic DNA was extracted from cecal contents using the E.Z.N.A.^®^ Soil DNA Kit (Omega Bio-tek, Norcross, GA, USA). The V3-V4 hypervariable region of the 16S rRNA gene was amplified using primers 338F (5′-ACTCCTACGGGAGGCAGCAG-3′) and 806R (5′-GGACTACHVGGGTWTCTAAT-3′). PCR products were purified and sequenced on the Illumina MiSeq platform (Majorbio Bio-Pharm Technology Co., Ltd., Shanghai, China). Raw sequencing data were processed using QIIME2 (version 2020.2). Reads were denoised and clustered into amplicon sequence variants (ASVs) or operational taxonomic units (OTUs) at 97% similarity. Alpha diversity indices (Sobs, Ace, Chao, Shannon, Heip) and beta diversity metrics (Bray–Curtis dissimilarity) were calculated. Principal coordinate analysis (PCoA) was performed to visualize community differences. Linear discriminant analysis effect size (LEfSe) was used to identify differentially abundant taxa among groups.

### 2.7. Statistical Analysis

Statistical analyses were performed using GraphPad Prism (version 8.02). Data are presented as mean ± standard error of the mean (SEM). Differences among multiple groups were assessed using one-way ANOVA followed by Tukey’s post hoc test. Statistical significance was set at *p* < 0.05, *p* < 0.01, and *p* < 0.001.

## 3. Results

### 3.1. QG Attenuates Heat Stress-Induced Jejunal Morphological Damage

**Figure 2:**
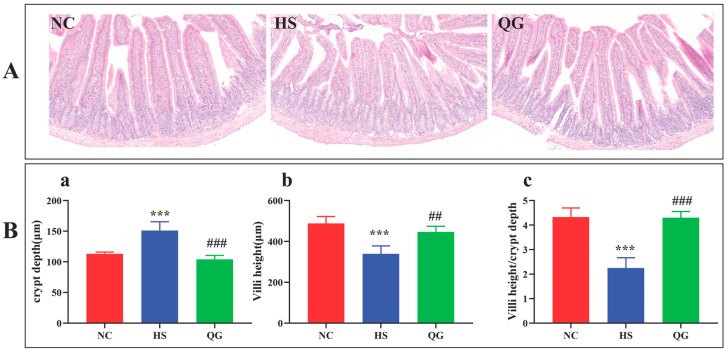
Effect of QG on intestinal tissue slices in mice. (**A**) Representative staining of jejunum sections (H&E × 100); (**B**) Differences in villi height and crypt depth in different treatment groups. (a) crypt depth, (b) Villi height, (c) Villi height to crypt depth ratio. *** *p*< 0.001, compared to normal controls (NC); ## *p* < 0.01, ### *p* < 0.001, compared to heat stress group (HS). NC, control; HS, heat stress; QG, quercetagetin.

H&E staining revealed that the HS group exhibited significant intestinal damage, characterized by disrupted villus architecture, villus shortening, and crypt deepening, compared to the NC group (Figure 2A). Morphometric analysis confirmed a significant reduction in villus height (* *p* < 0.01), a significant increase in crypt depth (## *p* < 0.01), and (** *p* < 0.001), consequently, a decreased villus height-to-crypt depth ratio (## *p* < 0.001) in the HS group (Figure 2B). QG pretreatment visibly ameliorated these histopathological alterations, preserving a more organized villus structure and significantly improving all three morphometric parameters (*p* < 0.05, *p* < 0.01, *p* < 0.001 vs. HS).

### 3.2. QG Reduces Stress Markers, Alleviates Oxidative Stress, and Suppresses Inflammation in the Jejunum of Heat-Stressed Mice

**Figure 3:**
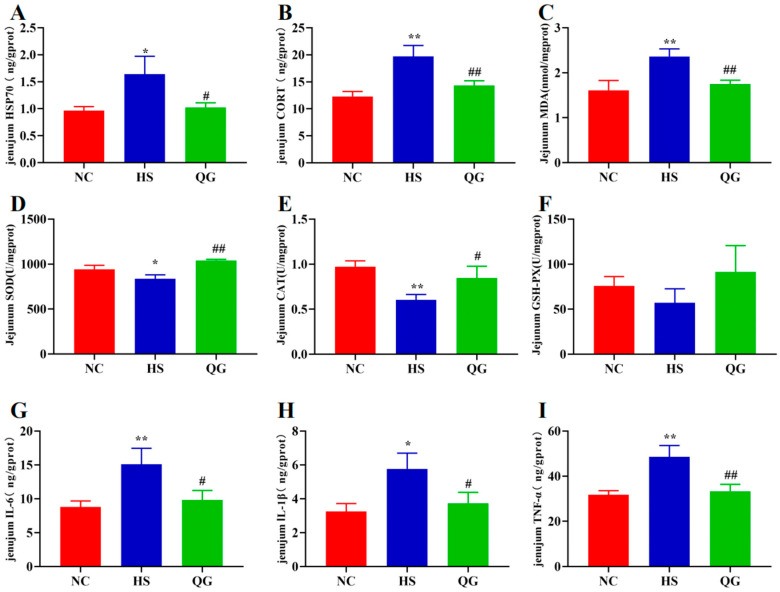
Illustrates the levels of malondialdehyde HSP70 (**A**), CORT (**B**), MDA (**C**), superoxide dismutase (SOD) (**D**), catalase (CAT) (**E**), glutathione peroxidase (GSH-PX) (**F**), IL-6 (**G**), IL-1β (**H**), and TNF-α (**I**) in mouse jejunal tissue. Statistical significance was determined using one-way analysis of variance (ANOVA), with * *p* < 0.05 and ** *p* <0.01 indicating significant differences compared to normal controls (NC), and # *p* < 0.05 and ## *p* < 0.01 indicating significant differences compared to the heat stress group (HS).

HS exposure significantly elevated the levels of HSP70 and CORT in the jejunum (* *p* < 0.05, ** *p* < 0.01 vs. NC), which were markedly reduced by QG treatment (# *p* < 0.05, ## *p* < 0.01 vs. HS) (Figure 3A,B). As shown in Figure 3C–F, HS induced significant oxidative stress, evidenced by increased MDA content (* *p* < 0.01 vs. NC) and decreased activities of SOD (* *p* < 0.05 vs. NC) and CAT (* *p* < 0.01 vs. NC). QG supplementation effectively countered this, significantly lowering MDA levels (## *p* < 0.01 vs. HS) and restoring SOD (## *p* < 0.01 vs. HS) and CAT (# *p* < 0.05 vs. HS) activities. GSH-Px activity showed no significant difference among the three groups.

### 3.3. QG Modulates the Expression of Genes Related to Heat Shock Response, Antioxidant Defense, and Intestinal Barrier Function

#### 3.3.1. Heat Shock Proteins

**Figure 4:**
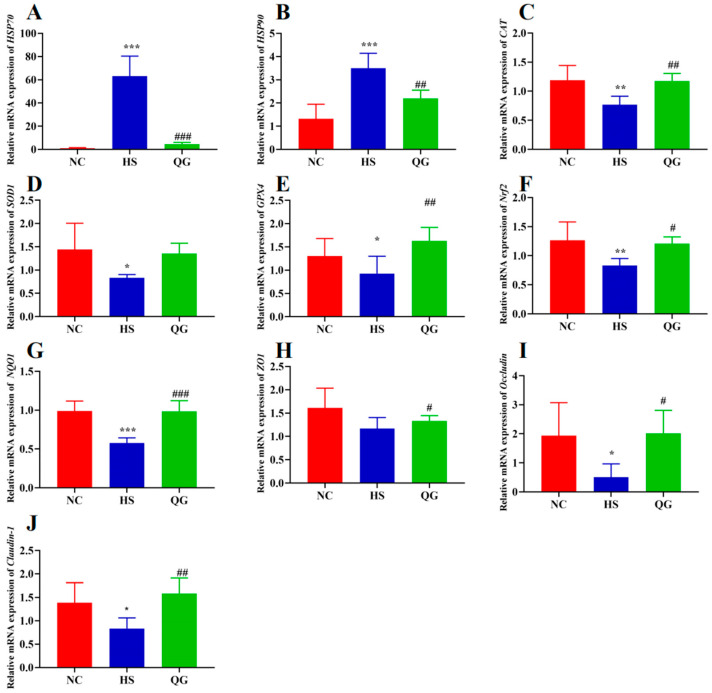
Effect of QG supplementation on heat stress-induced mRNA expression of antipyretic genes, antioxidant parameters, intestinal barrier and Nrf2 target genes in the mouse intestine. (**A**) *HSP70*, (**B**) *HSP90*, (**C**) *CAT*, (**D**) *SOD1*, (**E**) *GPX4*, (**F**) *Nrf2*, (**G**) *NQO1*, (**H**) *ZO1*, (**I**) *Occludin*, (**J**) *Claudin*. Significant differences were assessed by one-way analysis of variance (ANOVA), * *p* < 0.05, ** *p* < 0.01, *** *p* < 0.001, compared to normal controls (NC); # *p* < 0.05, ## *p* < 0.01, ### *p* < 0.001, compared to heat stress group (HS).

Consistent with the ELISA results, the mRNA expression levels of Hsp70 and Hsp90 were significantly upregulated in the HS group (* *p* < 0.01, ** *p* < 0.001 vs. NC). QG treatment significantly downregulated this HS-induced gene expression (## *p* < 0.01 vs. HS) (Figure 4A,B).

#### 3.3.2. Antioxidant Genes

QG supplementation significantly upregulated the mRNA expression of antioxidant enzymes *CAT* (## *p* < 0.01), *SOD1* (non-significant trend), and *GPX4* (## *p* < 0.01) compared to the HS group (Figure 4C–E). Notably, the expression of the master regulator *Nrf2* and its target gene *NQO1* was also significantly increased by QG treatment (## *p* < 0.01, ### *p* < 0.001 vs. HS) (Figure 4F,G), suggesting that QG may activate the *Nrf2* signaling pathway to enhance the antioxidant capacity.

#### 3.3.3. Tight Junction Proteins

HS exposure led to a disruption of the intestinal barrier at the transcriptional level, with reduced mRNA expression of tight junction proteins *ZO1* (non-significant), *Occludin* (* *p* < 0.05 vs. NC), and *Claudin*-1 (* *p* < 0.05 vs. NC) (Figure 4H–J). QG treatment significantly reversed this downregulation, restoring the expression of *ZO1* (# *p* < 0.05 vs. HS), Occludin (# *p* < 0.05 vs. HS), and *Claudin*-1 (## *p* < 0.01 vs. HS) to levels comparable to the NC group.

### 3.4. QG Modulates the Diversity and Composition of the Gut Microbiota in Heat-Stressed Mice

**Figure 5:**
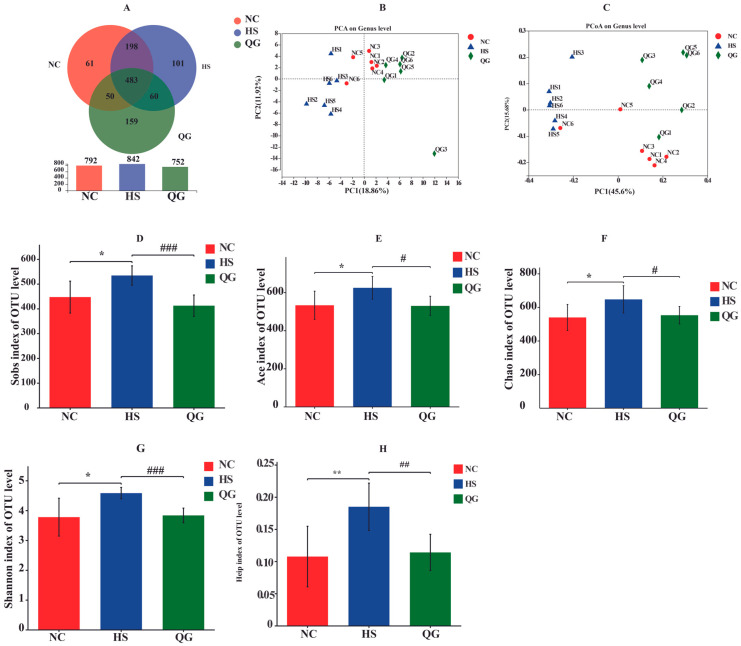
Effects of QG adinrantestinal microbiota inflammation in HS-induced intestinal mice. (**A**) A Venn diagram showing the overlap of the operational taxonomic units (OTUs) identified in the intestinal microbiota among three groups; (**B**) principal component analysis (PCA) plot of the gut microbiota; (**C**) principal coordinate analysis (PCoA) plot of the gut microbiota based on Bray–Curtis distances; (**D**) Sobs index of the OTU level; (**E**) Ace index of the OTU level; (**F**) Chaos index of the OTU level; (**G**) Shannon index of the OTU level; (**H**) Heip index of the OTU level; Data are presented as mean ± SEM. (*n* = 6 mice). * *p* < 0.05, ** *p* < 0.01, versus the normal control group (NC); # *p <* 0.05, ## *p <* 0.01, ### *p* < 0.001, versus the heat stress group (HS).

16S rRNA sequencing was conducted to evaluate the effects of QG on the gut microbial community. As shown in Figure 5, the analysis encompassed both alpha and beta diversity measures. First, a Venn diagram illustrated the distribution of operational taxonomic units (OTUs) among the groups, with 483 OTUs shared across all groups, and 61, 101, and 159 unique OTUs identified in the NC, HS, and QG groups, respectively (Figure 5A). Beta diversity analysis using principal component analysis (PCA) and PCoA based on Bray–Curtis distances revealed distinct clustering of the three groups, indicating that both HS and QG treatment significantly altered the overall microbial community structure (Figure 5B,C). For alpha diversity, the HS group exhibited significantly higher indices, including Sobs, Ace, Chao, Shannon, and Heip, compared to the NC group (* *p* < 0.05, ** *p* < 0.01 vs. NC), reflecting a marked shift in microbial community diversity (Figure 5D–H). QG treatment partially restored these indices toward normal levels, with significant differences observed when compared to the HS group (* *p* < 0.05, ** *p* < 0.01 vs. HS).

### 3.5. QG Alters the Taxonomic Composition of the Gut Microbiota

**Figure 6:**
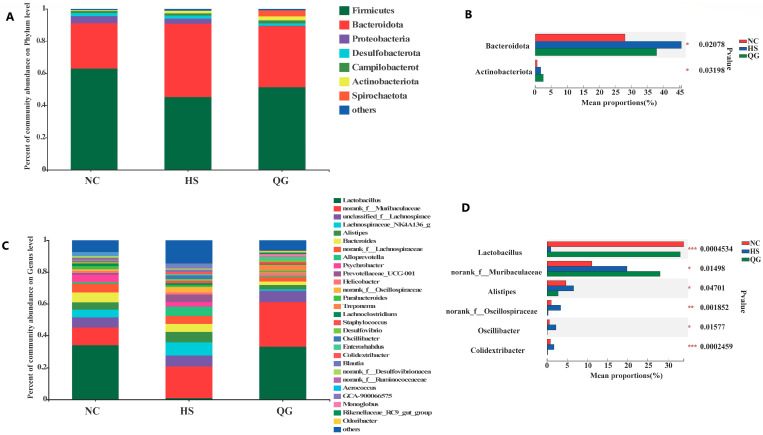
Effect of QG on the microbiota richness and distribution at the phylum and genus levels in HS-induced mice. Community composition of major bacteria at Phylum (**A**) and Genus (**C**) level; Average relative abundance of differential species in each group of mice at Phylum (**B**) and Genus (**D**), multi-group comparison with one-way ANOVA, followed by Fisher’s LSD test. * indicates statistically significant difference (*p* < 0.05), ** indicates highly significant difference (*p* < 0.01), *** indicates extremely significant difference (*p* < 0.001).

At the phylum level, Firmicutes and Bacteroidota were the dominant phyla (Figure 6A). HS exposure led to a significant increase in the relative abundance of Bacteroidota and a decrease in Firmicutes compared to the NC group (* *p* < 0.05) (Figure 6B). QG treatment significantly reversed this trend, decreasing Bacteroidota and increasing Firmicutes (# *p* < 0.05 vs. HS).

At the genus level, HS induced significant dysbiosis, characterized by a decreased relative abundance of the beneficial genus Lactobacillus and increased abundances of Alistipes, unclassified Oscillospiraceae, Oscillibacter, and Colidextribacter (* *p* < 0.05 vs. NC) (Figure 6C,D). QG supplementation effectively counteracted these HS-induced changes, significantly restoring the levels of Lactobacillus and reducing the potentially harmful genera (# *p* < 0.05 vs. HS). These results indicate that QG can partially restore the HS-induced dysbiosis of the gut microbiota.

### 3.6. Correlation Analysis Between Gut Microbiota and Physiological Parameters

**Figure 7:**
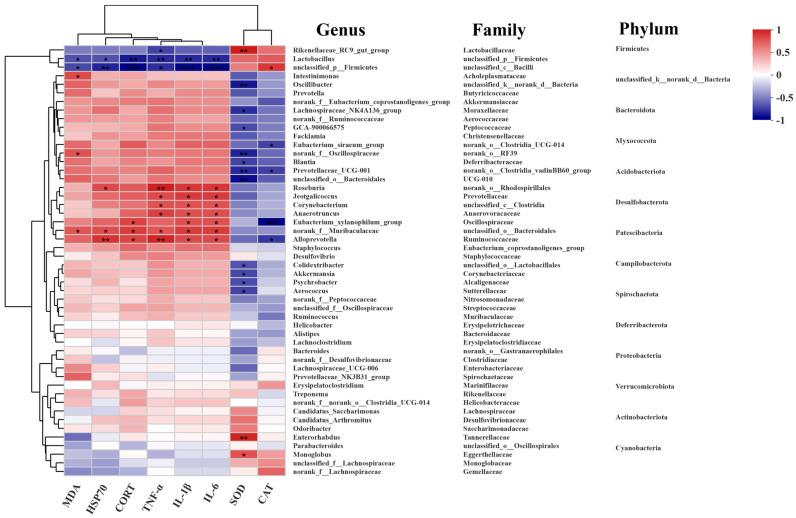
Spearman correlations between physicochemical indexes (HSP70, CORT, MDA, SOD, CAT, IL-6, IL-1βand TNF-α) and the communities of gut microbiota at the genus level. The Horizontal coordinate and Vertical coordinate are environmental factors and species, respectively. * *p* ≤ 0.05, ** *p* ≤ 0.01, *** *p* < 0.001.

Spearman’s rank correlation analysis was performed to explore the relationship between the relative abundance of the top 50 microbial genera and key physiological markers (Figure 7). Several genera showed significant correlations. Notably, Lactobacillus was positively correlated with the activities of antioxidant enzymes (SOD, CAT) and negatively correlated with pro-inflammatory cytokines (IL-1β, IL-6, TNF-α) and stress markers (HSP70, CORT). In contrast, genera enriched in the HS group, such as Oscillibacter and Colidextribacter, exhibited the opposite correlation pattern, being positively associated with inflammatory and stress markers and negatively with antioxidant enzymes. These correlations suggest a strong association between specific microbial taxa and the host’s physiological response to heat stress, although they do not imply causation.

## 4. Discussion

This study demonstrates that quercetagetin (QG) supplementation effectively mitigates heat stress (HS)-induced intestinal injury in mice. The protective effects of QG are multifaceted, involving (i) the enhancement of antioxidant capacity through the activation of the Nrf2 pathway and its downstream targets (NQO1, CAT, GPX4); (ii) the suppression of the inflammatory response, likely via inhibition of the NF-κB pathway; (iii) the preservation of intestinal barrier integrity by upregulating tight junction proteins (ZO1, Occludin, Claudin-1); and (iv) the modulation of the gut microbiota composition, partially restoring the abundance of beneficial bacteria like Lactobacillus while reducing potentially harmful taxa. These findings position QG as a promising candidate for a functional feed additive to combat the negative impacts of heat stress in animal production.

Our results align with previous studies showing that HS elevates pro-inflammatory cytokines (TNF-α, IL-1β, IL-6) and that natural compounds can suppress this response [37,38,39,40,41]. The observed reduction of these cytokines by QG is consistent with the anti-inflammatory effects reported for other polyphenols [42,43].

The Nrf2 signaling pathway is a central regulator of the cellular antioxidant defense [8,9,10]. The significant upregulation of Nrf2 and its target genes (NQO1, CAT, GPX4) in the QG-treated group strongly suggests that QG exerts its antioxidant effects, at least in part, through the activation of this pathway. This is further supported by the increased activities of SOD and CAT and the reduced lipid peroxidation (MDA) in the jejunum. This mechanism is analogous to that proposed for structurally similar compounds like quercetin or isoquercetin, which protect against oxidative stress via the Nrf2/HO-1 axis [44,45].

**Figure 8:**
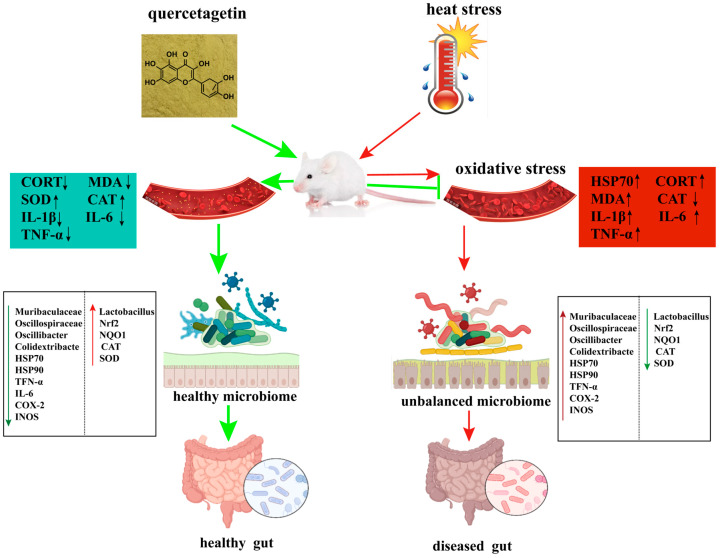
Proposed mechanism by which quercetagetin (QG) alleviates heat stress (HS)-induced intestinal injury. QG activates the Nrf2-ARE pathway, enhances antioxidant enzyme activity, reduces oxidative stress and inflammation, preserves tight junction integrity, and favorably modulates gut microbiota (e.g., increased Lactobacillus). Solid arrows indicate observed effects; dashed arrows indicate proposed but not directly tested pathways.

Interestingly, QG also modulated the heat shock response, downregulating the HS-induced overexpression of Hsp70 and Hsp90. While HSPs are initially protective, their sustained high expression can be a marker of severe cellular stress [46]. The ability of QG to restore Hsp70 or Hsp90 expression to near-normal levels may reflect an overall reduction in cellular stress, possibly as a consequence of enhanced antioxidant protection. This observation raises an intriguing question about the potential crosstalk between the Nrf2 and HSF1 pathways. Both pathways are stress-responsive and can be co-activated [11]. It is plausible that by alleviating oxidative stress, QG reduces the burden on the protein-folding machinery, thereby indirectly attenuating the HSF1-HSP response. We interpret the downregulation of Hsp70 and Hsp90 expression in the QG + HS group primarily as indicative of a diminished cellular stress burden. Given that heat shock proteins (HSPs) serve as sensitive biomarkers of proteotoxic stress, their reduced expression implies that QG pre-treatment effectively alleviated the initial protein-denaturing effects of heat stress, likely through the enhancement of antioxidant defenses via Nrf2 activation. This interpretation is further supported by the observed decrease in markers of oxidative damage, such as malondialdehyde (MDA), and inflammatory cytokines. Although we cannot entirely dismiss the possibility of a direct modulatory effect of QG on the HSF1 pathway, the current data are more congruent with the ‘stress alleviation’ hypothesis. Conversely, some compounds have been shown to directly interact with both pathways [12,13]. Future studies should investigate whether QG’s effects involve direct modulation of HSF1 or are an indirect consequence of improved redox status. A proposed model for QG’s mechanism of action is presented in Figure 8.

The preservation of intestinal barrier integrity by QG, evidenced by the upregulation of *Zo-1*, *Occludin*, and *Claudin-1*, is critical for preventing “leaky gut” and the subsequent systemic inflammation. This protective effect is likely a combined result of reduced oxidative damage and inflammation in the epithelial cells.

The gut microbiota is increasingly recognized as a key player in host health and stress response [47,48]. Our study shows that HS induces significant dysbiosis, increasing microbial diversity—which may reflect a destabilization of the community rather than a beneficial change—and altering the abundance of key taxa. The increase in potentially harmful genera like Oscillibacter and Colidextribacter, and the decrease in beneficial Lactobacillus, corroborates findings from other HS studies [49,50]. QG’s ability to partially restore this balance, particularly by enriching Lactobacillus, is noteworthy. This is consistent with recent findings in broilers, where dietary QG supplementation under high-stocking-density conditions significantly increased the abundance of beneficial bacteria such as Clostridia_vadinBB60_group_norank and modulated gut microbial composition to alleviate oxidative stress and mitochondrial dysfunction [51]. Lactobacillus species are known to enhance barrier function, modulate immunity, and produce antioxidant metabolites [52]. Furthermore, exopolysaccharides from related beneficial bacteria like Bifidobacterium have been shown to possess immunomodulatory capabilities [53]. The positive correlation between Lactobacillus and antioxidant enzymes, and its negative correlation with inflammatory markers, further supports its beneficial role in this context (Figure 7). The modulation of gut microbiota is therefore an important component of QG’s protective mechanism. It is plausible that this modulation results from a combination of both direct and indirect effects. On the one hand, quercetin glycoside (QG), a polyphenolic compound, may directly interact with the gut microbiota, functioning as a prebiotic or exerting selective antimicrobial effects. On the other hand, by mitigating oxidative stress and inflammation within the intestinal epithelium, QG enhances the host microenvironment, thereby promoting the proliferation of beneficial bacteria such as Lactobacillus. The strong correlations observed between Lactobacillus abundance and host antioxidant and anti-inflammatory markers (Figure 7) provide support for this indirect pathway. However, distinguishing between these two possibilities will require future studies using sterile fecal filtrates or in vitro microbial culture systems.

Our findings indicate that QG exhibits protective effects at a dosage of 100 mg/kg/day in mice; however, the applicability of this regimen to production animals, such as poultry or swine, necessitates careful evaluation. Utilizing allometric scaling based on body surface area, this murine dosage is approximately equivalent to a daily intake of 200–300 mg for a 2-kg broiler chicken, with proportionally greater amounts required for larger livestock. Although this may initially seem economically prohibitive, several factors suggest its practical feasibility. Firstly, QG can be efficiently extracted as a byproduct from marigold (*Tagetes erecta* L.) following lutein purification, providing a cost-effective and scalable source of raw material. Secondly, due to significant interspecies differences in gastrointestinal physiology and microbiota composition, species-specific dose-titration studies are crucial and may demonstrate that lower doses (e.g., 50–80 mg/kg feed) are sufficient to achieve similar protective effects in target animals. Third, the multifunctional advantages of QG, encompassing increased antioxidant capacity, diminished inflammation, enhanced intestinal barrier integrity, and modulation of gut dysbiosis, have the potential to lead to improved growth performance, reduced mortality, and decreased dependency on antibiotics, thereby compensating for the costs associated with feed additives. Future research should focus on dose–response assessments in pertinent farm species, alongside cost–benefit analyses, to develop practical and economically viable application protocols for QG as a functional feed additive under conditions of heat stress.

### Limitations and Future Directions

Several limitations should be acknowledged. First, the sample size (*n* = 6 per group) is relatively modest, especially for 16S rRNA-based microbiota analysis, and may affect the generalizability of the microbial findings. Second, the absence of a QG-only control group (QG under thermoneutral conditions) and a positive control group (e.g., vitamin C or quercetin) limits our ability to distinguish stress-specific effects from baseline physiological actions and to benchmark QG’s efficacy. Third, while our data suggest the involvement of the Nrf2 pathway, direct mechanistic proof using Nrf2-knockout mice or selective inhibitors (ML385) is required to confirm its necessity. Fourth, the correlation analysis between gut microbiota and host parameters does not imply causation. Future studies will address these limitations.

## 5. Conclusions

In summary, our study demonstrates that quercetagetin (QG) supplementation effectively alleviates heat stress-induced intestinal injury in mice. The protective mechanism is multifaceted, involving the possible activation of the Nrf2-mediated antioxidant defense, suppression of inflammation, preservation of the intestinal barrier, and partial restoration of a healthy gut microbiota composition. These findings provide a strong scientific rationale for further exploring QG as a functional feed additive to enhance intestinal health and resilience in livestock and poultry under heat stress conditions. Future research should focus on elucidating the precise molecular crosstalk between the Nrf2 and HSF1 pathways in response to QG and confirming its efficacy in target production animals.

## Figures and Tables

**Table 1:** Primer sequences used for RT-PCR.

Gene	Forward (5′-3′)	Reverse (5′-3′)
*HSP90*	GTTGCGGAGAAGGTGACAGTGATC	CTCCTTGATTCGCCGTTCTTCCAG
*HSP70*	CTGGCAATAAGCGAGCAGTGAGG	AATGCTGGCTTGCGTGGAAGAG
*SOD1*	AATGTGACTGCTGGAAAGGAC	CAATCCCAATCACTCCACAGG
*CAT*	ACATGGTCTGGGACTTCTGG	CAAGTTTTTGATGCCCTGGT
*GPX4*	AGTATGTGTGCTGCTC	CCAGTAATCACCAAGCCAATGC
*Nrf2*	CTGAACTCCTGGACGGGACTA	CGGTGGGTCTCCGTAAATGG
*NQO1*	AGGATGGGAGGTACTCGAATC	TGCTAGAGATGACTCGGAAGG
*ZO-1*	AGAAGATAGCCCTGCAGC	AGTCCGTAAGGAGATTCT
*Occludin*	GGTCAGGGAATATCCACC	ATTATATTCATCAGCAGC
*Claudin-1*	CAACGCGGGGCTGCAGCT	TTGTTTTCCGGGGACAGGA
*GAPDH*	CATGGCCTTCCGTGTTC	CCTGGTCCTCAGTGTAGC
*β-Actin*	CATCCGTAAAGACCTCTATGCCAAC	ATGGAGCCACCGATCCACA

## Data Availability

The original contributions presented in the study are included in the article, further inquiries can be directed to the corresponding authors.
